# Spectrum of Non-SARS-CoV-2 Respiratory Viruses Among Symptomatic Post-COVID Adults in a North Indian Tertiary Care Center: A 2022 Surveillance Study

**DOI:** 10.7759/cureus.104437

**Published:** 2026-02-28

**Authors:** Mahuya Roy, Subhabrata Sarkar, Vikrant Sharma, Ishani Bora, Ritin Mohindra, Supriya Padhy, Monika Sapra, Gursimran Kaur Mohi, Radha K Ratho

**Affiliations:** 1 Medical Microbiology, Postgraduate Institute of Medical Education and Research, Chandigarh, IND; 2 Virology, Postgraduate Institute of Medical Education and Research, Chandigarh, IND; 3 Internal Medicine, Postgraduate Institute of Medical Education and Research, Chandigarh, IND

**Keywords:** non-covid respiratory viruses, post-covid-19, respiratory symptoms, respiratory viruses, surveillance

## Abstract

Post-COVID, patients often suffer from long-term respiratory complications, contributing to increased susceptibility to secondary respiratory infections. Emerging surveillance studies have reported resurgence and altered seasonality of respiratory viruses following relaxation of non-pharmaceutical interventions, underscoring the need for surveillance of respiratory viruses in this cohort. The study included 108 post-COVID-19 patients (>18 years) presenting with respiratory symptoms from January to December 2022 in the Department of Medicine in Postgraduate Institute of Medical Education and Research (PGIMER), Chandigarh, a tertiary care facility in North India. Nasopharyngeal swabs, nasopharyngeal aspirates, and endotracheal aspirates were collected, and SARS-COV-2 RNA testing was performed on all, and samples negative for the same were screened for influenza A (H1N1, H3N2), influenza B, human bocavirus (hBoV), human adenovirus (hAdV), respiratory syncytial virus (RSV), human metapneumovirus (hMPV), and human rhinovirus (hRV). The statistical analysis was predominantly descriptive and was conducted using MS Excel 2024 (Microsoft Corp., Redmond, WA). The frequencies and percentages of categorical variables were summarized, and associations were evaluated using Fisher's exact test. Multivariable modeling was not conducted as a result of the descriptive study design and the small sample size. Among total patients, 20.37% were positive for respiratory viruses. Maximum positivity was observed with influenza A (37.5%), followed by hMPV (17%). A higher viral positivity rate was seen in females (p = 0.0361, Fisher’s exact test) and the geriatric age group (60 years, n = 40). Sequencing was done, and the circulating types were influenza A H1N1, influenza B Victoria type, RSV type A, and rhinovirus type A and C. This study describes the spectrum of respiratory viruses detected among symptomatic post-COVID patients with influenza, hMPV, and RSV as the predominant pathogens.

## Introduction

Respiratory infections, both acute and chronic, are exceedingly prevalent in both adults and children, placing a greater financial load on healthcare systems as well as increasing morbidity and mortality [1]. Respiratory viral infections have continued to be a substantial cause of acute respiratory illness (ARI) on a global scale, and their clinical significance was more pronounced during the SARS-CoV-2 pandemic [2]. The emergence of SARS-CoV-2 and the non-pharmaceutical interventions to curb the same resulted in a paradigm shift in the transmission patterns of respiratory viruses. Newer surveillance studies in the post-severe acute respiratory syndrome coronavirus 2 (SARS-CoV-2) era have documented a resurgence and altered seasonality of respiratory viruses as a result of the relaxation of non-pharmaceutical interventions [3]. Data describing the spectrum of non-SARS-CoV-2 respiratory viruses among symptomatic post-COVID adults in India remain limited, highlighting the need for targeted surveillance. We postulated that symptomatic post-COVID adults may be infected with multiple respiratory viruses.

The widespread implementation of non-pharmaceutical interventions during the COVID-19 pandemic, such as masking, physical distancing, and travel restrictions, has resulted in a marked decline in the circulation of common respiratory viruses worldwide. Nevertheless, the subsequent relaxation of these measures has been linked to atypical resurgence patterns, altered seasonality, and variations in the age distribution of respiratory viral infections [1,4-6].

ARIs remain one of the leading causes of outpatient visits and hospitalizations in all age groups, particularly in the winter months, as observed in North India [7-9]. To optimize diagnostic and clinical management strategies for patients with ARIs, it is imperative to possess an understanding of the epidemiological distribution of common respiratory viruses. The most commonly encountered non-influenza respiratory viruses that have drawn the attention of the scientific community are the respiratory syncytial virus (RSV), metapneumovirus, adenovirus, bocavirus, rhinovirus, coronavirus, and enterovirus [10].

In the post-COVID period, patients often suffer from long-term respiratory complications [11]. Studies suggest that SARS-CoV-2 infection may impair the CD8+ T cell responses [12]. Additionally, factors like post-COVID lung fibrosis, persistent immune dysregulation, and prolonged inflammation may contribute to increased susceptibility to secondary respiratory infections [13-15].

The majority of epidemiological studies conducted to date have focused on the clinical impact of respiratory viral infections in adults and the patterns of these infections during the COVID-19 pandemic. However, there is a scarcity of data on non-SARS-CoV-2 respiratory viruses among symptomatic adults in the post-COVID period. Targeted surveillance of non-SARS-CoV-2 respiratory viruses among symptomatic adults attending post-COVID follow-up clinics is therefore essential to define the epidemiological burden of circulating respiratory viruses, optimize diagnostic and clinical management strategies, and inform public health policies in the post-pandemic era. The objective of this study was to describe the spectrum of non-SARS-CoV-2 respiratory viruses in symptomatic post-COVID adults presenting to a tertiary care center using a surveillance-based approach.

## Materials and methods

Study population

A cross-sectional surveillance study was carried out at the Postgraduate Institute of Medical Education and Research (PGIMER), Chandigarh, India, to analyze the spectrum and clinical characteristics of other respiratory viruses among post-COVID-19 patients presenting with respiratory symptoms from January 2022 to December 2022 in the department of medicine. The study included 108 patients who had recovered from COVID-19 and presented with respiratory symptoms. Convenience sampling with feasibility-based recruitment was used. Post-COVID status was defined as individuals with prior RT-PCR-confirmed SARS-CoV-2 infection who had recovered clinically and tested negative for SARS-CoV-2 at recruitment. Patients were enrolled when they presented with persistent respiratory symptoms during follow-up, and there was no mandated time interval between recovery and recruitment. The study was approved by the Institutional Ethics Committee, PGIMER, Chandigarh, India (IEC letter no.: INT/IEC/2022/0001068 dated September 2, 2022). Written informed consent was obtained from all participants before sample collection. Patient demographic and clinical data were collected using a structured case record form.

Sample collection and processing

All adult post-COVID-19 patients (>18 years) presenting with respiratory symptoms during the study period were included. Patients with active SARS-CoV-2 infection or inadequate samples were excluded. The sample size was based on feasibility during the surveillance period. Nasopharyngeal swabs, nasopharyngeal aspirates, or endotracheal aspirates were collected after obtaining written informed consent. SARS-CoV-2 RNA testing was performed on all samples before they were processed to check for other respiratory viruses. Only samples negative for SARS-CoV-2 RNA were included in the current study. Screening for influenza A H1N1, influenza A H3N2, and influenza B by real-time PCR was performed using the TRUPCR® H1N1/H3N2 with Inf-B Kit (Kilpest India Ltd., Bhopal, India). Conventional PCR was performed for human bocavirus (hBoV) and the human adenovirus (hAdV), and conventional reverse transcriptase PCR following cDNA synthesis was done for RSV, human metapneumovirus (hMPV), and human rhinovirus (hRV) [16-19]. Ethidium bromide staining was used to identify the amplified DNA fragments on a 2% agarose gel, and they were then seen under a UV transilluminator. Laboratory testing was performed using validated multiplex RT-PCR assays with appropriate internal positive and negative controls in accordance with manufacturer instructions and laboratory quality assurance protocols. Sequencing PCR was performed using the commercially available BigDye® Terminator v3.1 Cycle Sequencing Kit (Thermo Fisher Scientific, Waltham, MA), followed by sequencing clean-up. The amplified products of the samples after purification were subjected to Sanger di-deoxy sequencing bidirectionally in Applied Biosystems 3500/3500xL Genetic Analyzer (Applied Biosystems, CA). Sequencing was conducted on samples with a sufficient viral load, and failures were attributed to inadequate template quality or quantity.

Data analyses

Statistical analysis was primarily descriptive and conducted using MS Excel (version 2024, Microsoft Corp., Redmond, WA). A p-value of <0.05 was considered statistically significant. No clinical severity scores or validated scales were applied in this study. Due to the modest sample size and descriptive surveillance design, analyses were primarily descriptive with Fisher’s exact test; multivariable modeling, regression analysis, and confidence interval estimation were not performed.

## Results

Clinical and demographic profile

**Figure 1:**
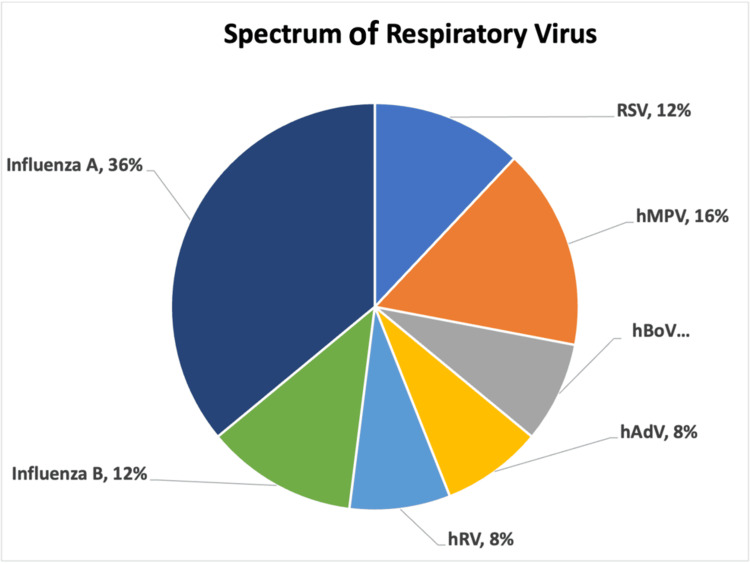
Spectrum of respiratory viruses among post-COVID-19 patients Each color represents a distinct viral pathogen. RSV: Respiratory syncytial virus; hMPV: Human metapneumovirus; hBoV: Human bocavirus; hAdV: Human adenovirus; hRV: Human rhinovirus.

In the current study, 20.37% (22/108) of patients were positive for respiratory viruses. Respiratory viral mono-infections were observed in 19 patients, and coinfections were observed in three patients. Among the 25 viruses identified in the PCR-positive patients, including coinfections, maximum positivity was observed for influenza A (n = 9; 36%), influenza A H1N1 (n = 4), and influenza A H3N2 (n = 5), followed by hMPV (n = 4; 17%), RSV (n = 3; 13%), and influenza B (n = 3; 13%) (Figure 1).

As a result of the identification of coinfections, the total virus count exceeded the number of patients. Males predominated among the 108 samples that were recruited for the study (M:F = 2:1), and the majority of participants (n = 40) were above the 60-year age category. The average age was 48.11 ± 24.61 years. Males had a higher prevalence in terms of viral positivity. Fever was the most frequently reported symptom (n = 85; 78.7%) (Table 1).

**Table 1:** Clinical and radiological features Values are expressed as n (%). P-values were calculated using Fisher’s exact test. Percentages in the first column were calculated using the total study population as the denominator (n = 108), while percentages in the positivity column were calculated using the respective subgroup as the denominator. Multiple symptoms per patient were allowed; therefore, totals may exceed 100%.

Symptoms/Signs	No. of patients screened (n = 108)	No. of positives among symptomatic patients	P-value
Fever	85 (78.7%)	16 (18.8%)	0.44
Abdominal pain	3 (2.8%)	1 (33.3%)	0.57
Vomiting	3 (2.8%)	1 (33.3%)	0.57
Cough	69 (63.9%)	15 (21.7%)	0.63
Sore throat	28 (25.9%)	5 (17.8%)	0.70
Nasal catarrh	12 (11.1%)	5 (41.6%)	0.052
Somnolence	2 (1.9%)	1 (50%)	0.29
Difficulty in breathing	69 (63.89%)	13 (18.8%)	0.59
Positive chest X-ray findings	47 (43.52%)	11 (23.4%)	0.49

Virus detection and sequencing results

Respiratory viral infection in post-COVID-19 patients was more common in women, and this difference was statistically significant (p = 0.0361, Fisher’s exact test), as 11 out of 34 women were positive, while 11 out of 74 men were positive. Despite the fact that males comprised the majority of the study population, females exhibited a substantially higher proportion of viral positivity. Prevalence was highest in the over-60 age group. There was no discernible association between any symptom or indication and viral positivity. Sequencing was performed, and the circulating types identified were influenza A H1N1, influenza B Victoria type, RSV type A, and rhinovirus types A and C (Table 2). The technical limitations and low viral load were presumably the reasons for the failure to sequence influenza A H3N2.

**Table 2:** Sequencing data RSV: Respiratory syncytial virus.

Identified virus	Lab ID	Circulating type
RSV	IB220	RSV A type
Rhinovirus	IB295; IB222	Rhinovirus type C; rhinovirus type A
Influenza B	IB453; IB461; IB482	Influenza B Victoria type
Influenza A H1N1	IB460	Influenza A H1N1
Influenza A H3N2	IB513	Was not amenable to sequencing

## Discussion

The recent pandemic has increased the burden of respiratory problems on the healthcare system, especially primary care. Additionally, the long-term effect will continue to have an impact on how respiratory treatment is delivered for months or even years to come [20]. In the post-pandemic world, the resurgence of non-SARS-CoV-2 respiratory viruses is slowly becoming a growing concern, mainly due to the relaxation of pandemic control measures. The objective of this study was to explore the spectrum of respiratory infections in post-COVID-19 patients with respiratory symptoms in a tertiary care center in North India.

The study is primarily composed of male patients (M:F = 2:1); however, females exhibited a greater viral positivity rate, which was statistically significant (p = 0.0361). This finding challenges previous studies showing that men are more susceptible to respiratory tract infections (RTIs) and severe disease progression. The majority of the literature indicates that men overall have a higher risk of hospitalization, ICU admission, and mortality from respiratory infections than women, possibly due to biological and behavioral factors such as smoking, occupational exposure, and differences in the immune response [21,22]. However, a few studies highlight the fact that female patients have a significantly higher incidence of respiratory symptoms compared with male patients. The higher viral positivity observed among females should be interpreted with caution. Although previous research has suggested that biological and sociocultural factors can influence the risk of respiratory infections, the present study did not evaluate these mechanisms, and, as a result, they cannot be inferred [23,24].

We observed a higher proportion of elderly individuals among the recruited participants, which is indicative of the demographic profile of patients presenting with post-COVID respiratory symptoms. The majority of participants in our study (n = 40) were above the age of 60. In this study, 12% (13/108) of patients expired, the majority of whom were above 60 years (7/13), which reinforces the fact that the geriatric age group has been identified as a vulnerable population for severe respiratory tract infection. The mortality findings were descriptive and were not analyzed for their potential correlation with viral positivity. Defects in cell-mediated and humoral immunity, alcohol and tobacco use, the presence of polypharmacy, weakened immune systems, and other comorbidities, along with concomitant therapies, may all contribute to the higher occurrence of RTIs in older patients [25,26].

The signs and symptoms of RTIs are well-known and recognizable. Fever was the most prevalent symptom in our study as well, followed by coughing and breathing problems; however, we were unable to detect any connection between the symptoms and virus positivity. This result confirms that it is still difficult to distinguish between bacterial and viral illnesses based just on symptoms and that molecular diagnostics are required for precise identification. Since inflammatory mediators such as prostaglandins and cytokines play a part in symptom manifestation, it might be challenging to differentiate viral infections solely by clinical presentation, according to several studies [27]. The limitation of syndromic diagnosis in RTIs is underscored by the absence of a distinct association between specific clinical symptoms and viral positivity in this study [28]. Although a near-significant association between viral positivity and nasal catarrh was observed (p = 0.052), that association may warrant further investigation in larger cohorts. A common consequence of the overlapping clinical presentations of viral and bacterial infections is the use of empirical antimicrobials, which exacerbates antimicrobial resistance [29]. Molecular diagnostics are therefore essential for the precise identification of viral etiologies, which in turn enables targeted therapy, rational antimicrobial use, and appropriate infection control measures, particularly in the post-COVID-19 environment.

While RSV has been historically recognized as a predominant viral pathogen in RTIs, several studies, including ours, have identified influenza as the leading cause of respiratory viral infections in adults. In our study, 20.4% of the patients tested positive for a respiratory virus, with influenza A (H3N2 and H1N1) being the most common (36%), followed by hMPV (16%) as well as RSV and influenza B (12% each). RSV has been identified as the primary viral culprit in RTIs in several investigations conducted in North India [9,30], although some studies have reported influenza virus as the most prevalent [31]. These results illustrate the viral spectrum that was observed in this cohort, underscoring the necessity of ongoing surveillance to monitor emergent trends.

Post-COVID-19 patients are a clinically vulnerable group, characterized by frequent healthcare visits and ongoing respiratory morbidity [32]. Our study highlights the need for enhanced viral surveillance in post-COVID-19 patients presenting with respiratory symptoms. Given the resurgence of non-COVID-19 virus in the community, continuous monitoring, timely vaccination, and molecular diagnostics should be prioritized to tackle future outbreaks. The study also highlights the importance of influenza vaccination in the elderly, especially those with comorbidities, to reduce morbidity and mortality associated with respiratory tract infection. The integration of multiplex respiratory viral testing into post-COVID follow-up clinics may facilitate clinical decision-making based on symptoms. A pragmatic diagnostic approach may be represented by selective multiplex PCR testing, which is guided by symptom-based triage in resource-constrained settings. The study's primary strengths are the year-long surveillance of symptomatic post-COVID adults in a clinically relevant tertiary care setting and the molecular confirmation of respiratory viruses with sequencing when feasible.

This study has several limitations. The cross-sectional descriptive design and convenience sampling in a single-center tertiary care setting may introduce selection bias and restrict the generalizability of the study to the community. The statistical efficacy was limited by the small sample size, which also prevented the use of multivariable adjustment to account for potential confounders, such as age and sex. The absence of a comparator group and the absence of longitudinal follow-up restrict the ability to infer causal associations or temporal epidemiological changes. Information regarding vaccination status, severity of prior COVID-19 infection, smoking status, occupational exposures, and comorbidities was not collected, which limited the interpretation of demographic and clinical associations. This study did not assess immunological mechanisms, and the mortality outcomes were descriptive without causal attribution. Furthermore, the inclusion of solely symptomatic post-COVID individuals may result in an underestimation of the overall burden of respiratory viral infections. To more accurately define the epidemiological patterns and clinical impact of non-SARS-CoV-2 respiratory viruses in the post-COVID era, future research that integrates asymptomatic controls, longitudinal follow-up, and larger multicenter cohorts is required.

## Conclusions

This study describes the spectrum of respiratory viruses detected among symptomatic post-COVID adults, with influenza, hMPV, and RSV representing the most frequently identified pathogens. The sample population was significantly composed of older individuals, which is indicative of the demographic profile of patients who demonstrate post-COVID respiratory symptoms. Despite the fact that males comprised the majority of the recruited participants, females exhibited a higher proportion of viral positivity. The significance of ongoing respiratory virus surveillance is underscored by these findings, which describe viral circulation within this clinical cohort. The integration of multiplex respiratory viral testing into post-COVID follow-up clinics may facilitate symptom-guided diagnostic strategies, particularly in resource-limited environments, and may provide valuable insights for future larger multicenter studies.

## References

[1] Niederman MS, Torres A (2022). Respiratory infections. Eur Respir Rev.

[2] Manno M, Pavia G, Gigliotti S (2025). Respiratory virus prevalence across pre-, during-, and post-SARS-CoV-2 pandemic periods. Viruses.

[3] Hong S, Zhu M, Huang Y (2025). Post-pandemic resurgence of respiratory virus infections: age and department-specific patterns in a Chinese tertiary hospital (2020-2024). BMC Infect Dis.

[4] Nichols WG, Peck Campbell AJ, Boeckh M (2008). Respiratory viruses other than influenza virus: impact and therapeutic advances. Clin Microbiol Rev.

[5] Pavia AT (2011). Viral infections of the lower respiratory tract: old viruses, new viruses, and the role of diagnosis. Clin Infect Dis.

[6] Huang QS, Wood T, Jelley L (2021). Impact of the COVID-19 nonpharmaceutical interventions on influenza and other respiratory viral infections in New Zealand. Nat Commun.

[7] Nieto-Rivera B, Saldaña-Ahuactzi Z, Parra-Ortega I (2023). Frequency of respiratory virus-associated infection among children and adolescents from a tertiary-care hospital in Mexico City. Sci Rep.

[8] Ramani VK, Pattankar J, Puttahonnappa SK (2016). Acute respiratory infections among under-five age group children at urban slums of Gulbarga city: a longitudinal study. J Clin Diagn Res.

[9] Waghmode R, Jadhav S, Nema V (2021). The burden of respiratory viruses and their prevalence in different geographical regions of India: 1970-2020. Front Microbiol.

[10] Kesson AM (2007). Respiratory virus infections. Paediatr Respir Rev.

[11] Acharya S, Akram A, Kodali A, Donovan S, Gupta SS, Kodali L (2023). Post COVID-19 pulmonary complications in outpatient setting: Insights from a cross-sectional study in a rural academic hospital. Am J Emerg Med.

[12] Gao F, Mallajosyula V, Arunachalam PS (2023). Spheromers reveal robust T cell responses to the Pfizer/BioNTech vaccine and attenuated peripheral CD8(+) T cell responses post SARS-CoV-2 infection. Immunity.

[13] Ambardar SR, Hightower SL, Huprikar NA, Chung KK, Singhal A, Collen JF (2021). Post-COVID-19 pulmonary fibrosis: novel sequelae of the current pandemic. J Clin Med.

[14] Achkar M, Jamal O, Chaaban T (2022). Post-COVID lung disease(s). Ann Thorac Med.

[15] Singh SJ, Baldwin MM, Daynes E (2023). Respiratory sequelae of COVID-19: pulmonary and extrapulmonary origins, and approaches to clinical care and rehabilitation. Lancet Respir Med.

[16] Bharaj P, Sullender WM, Kabra SK (2009). Respiratory viral infections detected by multiplex PCR among pediatric patients with lower respiratory tract infections seen at an urban hospital in Delhi from 2005 to 2007. Virol J.

[17] Bouscambert-Duchamp M, Lina B, Trompette A, Moret H, Motte J, Andréoletti L (2005). Detection of human metapneumovirus RNA sequences in nasopharyngeal aspirates of young French children with acute bronchiolitis by real-time reverse transcriptase PCR and phylogenetic analysis. J Clin Microbiol.

[18] Wisdom A, Kutkowska AE, Leitch ECM, Gaunt E, Templeton K, Harvala H, Simmonds P (2009). Genetics, recombination and clinical features of human rhinovirus species C (HRV-C) infections; interactions of HRV-C with other respiratory viruses. PLoS One.

[19] Kesebir D, Vazquez M, Weibel C, Shapiro ED, Ferguson D, Landry ML, Kahn JS (2006). Human bocavirus infection in young children in the United States: molecular epidemiological profile and clinical characteristics of a newly emerging respiratory virus. J Infect Dis.

[20] Bostock B (2021). Gender and respiratory conditions: are men at increased risk?. Trends in Urology & Men’s Health.

[21] Fine MJ, Smith MA, Carson CA, Mutha SS, Sankey SS, Weissfeld LA, Kapoor WN (1996). Prognosis and outcomes of patients with community-acquired pneumonia. A meta-analysis. JAMA.

[22] Falagas ME, Mourtzoukou EG, Vardakas KZ (2007). Sex differences in the incidence and severity of respiratory tract infections. Respir Med.

[23] Groeneveld JM, Ballering AV, van Boven K, Akkermans RP, Hartman TCO, Uijen AA (2020). Sex differences in incidence of respiratory symptoms and management by general practitioners. Fam Pract.

[24] Pinkerton KE, Harbaugh M, Han MK (2015). Women and lung disease: sex differences and global health disparities. Am J Respir Crit Care Med.

[25] Hoyert DL, Kung HC, Smith BL (2005). Deaths: preliminary data for 2003. Natl Vital Stat Rep.

[26] Akhtar A, Hassali MA, Zainal H, Ali I, Iqbal MS, Khan AH (2021). Respiratory-tract infections among geriatrics: prevalence and factors associated with the treatment outcomes. Ther Adv Respir Dis.

[27] Kuchar E, Miśkiewicz K, Nitsch-Osuch A, Szenborn L (2015). Pathophysiology of clinical symptoms in acute viral respiratory tract infections. Adv Exp Med Biol.

[28] Broaddus VC, Mason RJ, Ernst JD (2016). Murray and Nadel's Textbook of Respiratory Medicine. Textbook of Respiratory Medicine. Elsevier.

[29] Ahmed SK, Hussein S, Qurbani K, Ibrahim RH, Fareeq A, Mahmood KA, Mohamed MG (2024). Antimicrobial resistance: impacts, challenges, and future prospects. J Med Surg Public Health.

[30] Sapra M, Kirubanandhan S, Kanta P, Ghosh A, Goyal K, Singh MP, Ratho RK (2022). Respiratory viral infections other than SARS CoV-2 among the North Indian patients presenting with acute respiratory illness during the first COVID-19 wave. Virusdisease.

[31] Kumar R, Dar L, Amarchand R (2021). Incidence, risk factors, and viral etiology of community-acquired acute lower respiratory tract infection among older adults in rural north India. J Glob Health.

[32] Pavli A, Theodoridou M, Maltezou HC (2021). Post-COVID syndrome: incidence, clinical spectrum, and challenges for primary healthcare professionals. Arch Med Res.

